# What Influences Chinese Adolescents’ Choice Intention between Playing Online Games and Learning? Application of Theory of Planned Behavior with Subjective Norm Manipulated as Peer Support and Parental Monitoring

**DOI:** 10.3389/fpsyg.2017.00589

**Published:** 2017-04-18

**Authors:** Jia Wang, Ru-De Liu, Yi Ding, Ying Liu, Le Xu, Rui Zhen

**Affiliations:** ^1^Beijing Key Laboratory of Applied Experimental Psychology, Institute of Developmental Psychology, School of Psychology, Beijing Normal UniversityBeijing, China; ^2^Graduate School of Education, Fordham University, New YorkNY, USA

**Keywords:** online games, learning, choice intention, theory of planned behavior, manipulated subjective norm

## Abstract

This study investigated how and why Chinese adolescents choose between playing online games and doing homework, using the model of the theory of planned behavior (TPB) in which the subjective norm was manipulated as two sub-elements (peer support and parental monitoring). A total of 530 students from an elementary school and a middle school in China were asked to complete the measures assessing two predictors of TPB: attitude and perceived behavioral control (PBC). Next, they completed a survey about their choice intention between playing an online game and doing homework in three different situations, wherein a conflict between playing online games and doing homework was introduced and subjective norm was manipulated as peers supporting and parents objecting to playing online games. The results showed that adolescents’ attitude and PBC, as well as the perception of obtaining or not obtaining support from their peers and caregivers (manipulated subjective norm), significantly influenced their choice intention in online gaming situations. These findings contribute to the understanding of the factors affecting adolescents’ online gaming, which has been a concern of both caregivers and educators. With regard to the theoretical implications, this study extended previous work by providing evidence that TPB can be applied to analyze choice intention. Moreover, this study illuminated the effects of the separating factors of subjective norm on choice intention between playing online games and studying.

## Introduction

In recent years, online gaming has become one of the most popular global leisure activities. According to the China Internet Network Information Center [CNNIC] (2014), 368 million Chinese people played online games in 2014, and the numbers are predicted to increase. More importantly, adolescents comprise the highest proportion of Chinese online game players (China Internet Network Information Center [CNNIC], 2014). This unprecedented number of adolescent players has received the attention of researchers. Some researchers have focused on the effects of online gaming on individuals’ mental health, cognitive development, academic achievement, and interpersonal relationships (e.g., Van Reijmersdal et al., 2010; Zhong, 2011; Lin et al., 2012; Kowert et al., 2015). Others have investigated the factors that promote adolescents to play online games (e.g., Kim et al., 2008; Huang and Hsieh, 2011; Lee, 2015).

### How Chinese Adolescents’ Choose between Playing Online Games and Studying: Using TPB as Theoretical Frame

One of the greatest concerns is how much time adolescents spend playing online games instead of learning on a daily basis. How much time per week that individuals spend playing online games is a standard method of diagnosing online game addiction (Charlton and Danforth, 2007). Adolescents’ participation in online games can take up the time they are supposed to spend on learning, which leads to the significant negative correlation between playing online games and academic performance (e.g., Suhail and Bargees, 2006; Jiang, 2014). To understand how adolescents distribute their time between online games and learning tasks, it is worthwhile to investigate how adolescents make choices between playing online games and learning. The present study attempted to investigate this issue by utilizing two theoretical frameworks: the theory of planned behavior (TPB; Ajzen, 1991) and ecological systems theory (EST; Bronfenbrenner, 1979; Bronfenbrenner and Evans, 2000).

The TPB (Ajzen, 1991) has postulated that the possibility of engaging in one behavior can be predicted by an individual’s intention and that this intention is determined by attitude, perceived behavioral control (PBC), and the subjective norm. Attitude reflects whether one likes or dislikes engaging in an activity (Lee and Tsai, 2010; Hong et al., 2011). PBC is defined as the summation of an individual’s beliefs about behavioral control (Russell and William, 2007; Lu and Wang, 2008; Hong et al., 2011). Subjective norm is defined as one’s perceptions regarding whether important others believe that he/she should perform the certain behavior (Baker and White, 2010). According to TPB, individuals are more likely to perform a particular behavior when they evaluate this behavior more favorably, perceive volitional control over this behavior, and perceive that important others support them (Ajzen and Fishbein, 1980; Ajzen and Madden, 1986; Ajzen, 1987; Lee and Tsai, 2010).

Theory of planned behavior has been proven to be effective in predicting network behaviors and technology use (e.g., Baker and White, 2010; Teo and Lee, 2010; Chen, 2013). In a recent study, Lee and Tsai (2010) first applied TPB to explain why people continued to play online games. Their research model included TPB along with the technology acceptance model and flow theory. As expected, they found that all three predictors of TPB–attitude, PBC, and subjective norm–positively related to participants’ continued intention to play online games (Lee and Tsai, 2010). Although these findings were encouraging, more than half of the participants were between 20 and 40 years of age (Lee and Tsai, 2010), and thus we cannot directly apply this conclusion to adolescents. Another study investigated the predictive capacity of TPB on playing a particular online moral game, using elementary school students as participants (Hong et al., 2011). Hong et al. (2011) replicated the findings of Lee and Tsai (2010) and extended their study to adolescents. However, they drew their conclusions from studying a particular game named “To do or not to do.” It is necessary to verify whether these conclusions can be expanded to the whole field of online games.

Both studies made initial efforts to explain online game behavior and intention using the framework of TPB. However, the researchers focused their studies on the case of online gaming in which the players did not have other tasks except playing online games. Chinese elementary and middle school students have to spend considerable time on study even after school. If they want to play online games, they must consider how to allocate their time. Thereupon, the question arises as to what influences Chinese adolescents’ choice intention when they encounter the scheduling conflict between playing online games and completing learning tasks. TPB, which is widely used to predict adolescents’ behavioral intentions, might be appropriate to explain their choice intention regarding online games. However, previous studies used TPB only to explain common online gaming behavior and intention, as mentioned above, ignoring the conflict between playing and learning. To better understand adolescents’ online-game behaviors and to extend the previous work of TPB, the present study investigated how and why adolescents choose between playing online games and completing learning tasks, using a TPB framework. Specifically, the predictive roles of attitude, PBC, and subjective norm on adolescents’ choice intention regarding online games, as outlined by TPB, were investigated in this study, wherein the participants faced a conflict choice between playing online games and doing homework.

### Extending TPB with Manipulating Subjective Norm as Perceived Peer Support and Parental Monitoring

Although the efficiency of TPB has been supported by several studies (e.g., Baker and White, 2010; Lee and Tsai, 2010; Teo and Lee, 2010; Hong et al., 2011; Chen, 2013), some differing views should be acknowledged. Ajzen (1991) and Armitage and Conner (2001) found that the predictive power of subjective norm on behavioral intention was lower than the predictive power of attitude and PBC. This weaker subjective norm-intention link might be due to an insufficient definition and measurement of subjective norm (Terry and Hogg, 1996; Terry et al., 1999). Terry and Hogg (1996) suggested that perceptions of the influence of important others could not fully reflect the role of social influence. Chang et al. (2014) further clarified this point of view. They argued that if peers or friends did not have enough knowledge of a certain behavior to make a constructive suggestion, it would thus become difficult to explain human behavior by only relying on measuring the subjective norm as the single source of social influence (Chang et al., 2014). The results of Baker and White (2010) further supported these findings. They found that subjective norm did not significantly influence social networking site use intention when controlling for the effect of group norms (Baker and White, 2010). Consequently, it might be inferred that objective consideration of important others’ support of or objection to a behavior may explain the role of social influence more comprehensively than measuring perceived social norms or normative pressure in predicting one’s behavior and behavioral intention.

Notably, most measures of subjective norm merely focus on the influence of “important others” generally; for example, “Most people who are important to me would want me to socialize online” (e.g., Baker and White, 2010; Chang et al., 2014; Heirman et al., 2016; Jafarkarimi et al., 2016; Kim et al., 2016). However, the different beliefs of important others are ignored. Particularly, it is difficult to determine whether important others support a certain behavior, if some (e.g., peers) approve this behavior whereas others (e.g., parents or teachers) object to this behavior. In this dilemma, the measure of subjective norm is ambiguous, which cannot fully reflect the real perceived social norm. This may be one of the reasons that led to previous inconsistent conclusions regarding the effect of subjective norm on behavioral intention. Thus, it is of great value to further clarify the relation between subjective norm and intention by applying other methods rather than measuring subjective norm traditionally.

Within the Chinese cultural context, attitudes toward online games of adults and adolescents are conflicting. In China, most online gamers are adolescents (China Internet Network Information Center [CNNIC], 2014). However, adults, especially parents, often object to adolescents’ playing online games. A survey showed that nearly half of Chinese parents believed that playing online games is harmful to adolescents (Liu et al., 2015). According to EST (Bronfenbrenner, 1979, 1994; Bronfenbrenner and Evans, 2000), both parents and peers are important others and have substantial impact on adolescents’ behavior and development. This means that Chinese adolescents might perceive conflicting opinions from their important others (parents and peers) regarding their online gaming. Imagine a very common phenomenon for a child in China: the child’s friends ask him/her to play online games with them after school, while the child’s parents do not give permission for him/her to play. It would be very interesting to investigate what adolescents intend to do and how subjective norm influences their intentions of playing online games in this case, wherein their peers support playing but their parents object to playing. However, as argued above, it cannot fully reflect one’s perceptions regarding whether important others believe that he/she should play online games using previous measures in this dilemma. In other words, participants may perceive that their peers support their playing online games, at the same time their parents object to their playing, but they cannot rate both a 1 (*strongly disagree*) and a 5 (*strongly agree*) on a single Likert scale when being asked whether important others want them to play online games. To address this issue and to better understand the effect of subjective norm on online gaming choice intentions, the present study changed measuring subjective norm into manipulating subjective norm using a scenario-based instrument. Specifically, this study manipulated parent and peer norms, which are components of subjective norm (Cicognani et al., 2016). Additionally, in this study, parents and peers held different opinions regarding their support of participants’ playing online games.

### The Present Study

In sum, the present study aimed to investigate adolescents’ decision-making intention regarding playing online games or doing homework by creating specific learning situations. The model of TPB was used to explain their choice intention. Based on TPB and previous literature, it was supposed that:

H1: Attitude toward online games would positively predict adolescents’ online game-playing intention.
H2: PBC would positively predict adolescents’ online game-playing intention.
  Furthermore, unlike the traditional measurement of subject norm, the present study created a joint peer support situation and parental monitoring situation to manipulate subjective norm. Specifically, the manipulation included three situations: the first was the general learning situation (the control condition), wherein there was no manipulation of subjective norm; the second was the peer support situation, wherein the subjective norm was manipulated such that important others supported participants’ playing online games; the last was the parental monitoring situation, wherein the subjective norm was manipulated such that important others did not want them to play. Thereupon, the effect of subjective norm on choice intention can be illuminated through comparing the difference in the participants’ choice intention in these situations. The following hypotheses are presented:
H3: The number of participants who intended to choose playing online games would significantly increase in the peer support situation in comparison to the general learning situation.
H4: The number of participants who intended to choose playing online games would significantly decrease in the parental monitoring situation in comparison to the peer support situation.

## Materials and Methods

### Participants

This study was approved by the Research Ethics Committee of Beijing Normal University and the principals of the participating schools. Informed consent was obtained from all individual participants included in the study. A total of 530 students (male: *n* = 268; female: *n* = 262) in Beijing volunteered to participate in this study; 48.49% of the participants were elementary school students (Grade 5, *n* = 257), and the rest were middle school students (Grade 7, *n* = 273).

### Procedure

Participants were first asked to complete two questionnaires to assess their online game attitude and PBC. Then, the participants were asked to choose whether they intended to do homework or play online games in three different learning situations. Standardized instructions were provided.

The first situation was a general learning situation, which is very common for students in primary and secondary school. This situation was created as a control condition to explore the influence of the manipulated subjective norm on adolescents’ decision-making intentions regarding online games. The instruction of this scenario was as follows: Imagine a situation that may happen in the future, wherein you have not finished your homework, which will be checked by your teacher tomorrow morning, but you want to play online games. After having read the description of this scenario and imagined the situation, the participants were asked to answer the question: “Which one do you intend to select to do in this situation, playing online games or doing homework?” Following this, the other two situations that manipulated subjective norm (peer support situation and parental monitoring situation) were introduced in sequence with the following instructions: (1) At this moment, your friends invite you to play online games with them. (2) Although your friends want you to play online games with them, your parents think that playing online games might take up your homework time and that you should not play now. In each of the two cases, participants were asked to again decide their choice intention between doing homework and playing online games.

### Measures

#### Attitude

Participants reported their attitude toward online gaming using Lee and Tsai’s (2010) three-item measure. The term “playing X on this website” in the original scale was replaced by “playing online games” in this study. The three items were (a) “I think playing online games is good for me,” (b) “I think playing online games is a good leisure activity,” and (c) “I have a positive opinion about playing online games.” Participants responded on a 5-point Likert scale ranging from 1 (*strongly disagree*) to 5 (*strongly agree*).

#### Perceived Behavioral Control

Perceived behavioral control was measured by five items adapted from Lee and Tsai (2010) and Aboelmaged and Gebba (2013). The five items were (a) “I am able to play online games without help,” (b) “Playing online games is entirely within my control,” (c) “I have the resources to play online games,” (d) “I have the knowledge to play online games,” and (e) “I have the ability to play online games.” Responses were rated on a 5-point Likert scale ranging from 1 (*strongly disagree*) to 5 (*strongly agree*).

The alphas of the attitude measure and the PBC measure were 0.71 and 0.75 in this study, respectively. The confirmatory factor analysis of the measurement model was carried out, and the overall fit index was as follows: *χ^2^/df* = 2.396, CFI = 0.982, TLI = 0.968, RMSEA = 0.051. These results showed that the two measures had good reliability and construct validity.

### Data Analyses

The analyses of data were conducted applying SPSS 20.0 software. First, the descriptive statistics were conducted. Twenty-two cases with missing values on continuous variables (attitude and PBC) were imputed using the EM algorithm, and the Little’s MCAR test indicated that the data were missing completely at random (χ^2^ = 87.93, *p* = 0.112). Series mean was adopted to handle the missing data. The sample characteristics and descriptive statistics are illustrated in **Table 1**. The results showed that there were no cases with extreme *z* scores, which indicated that there were no univariate outliers. Multivariate outliers within the groups were sought applying SPSS Regression software with choice intention in the three situations as the dependent variable, respectively, and with grade, gender, attitude, and PBC as independent variables (Tabachnick and Fidell, 2006). The values of Mahalanobis distance ranged from 1.92 to 18.34 (lower than 18.47, *p* >0.001), which indicated that no cases were identified as multivariate outliers. The two measured variables (attitude and PBC) obtained values ranging from 0.24 to 0.27 for skewness, and from -0.66 to -0.47 for kurtosis, which were accepted for the univariate normality assumption of the maximum likelihood estimation (Finney and DiStefano, 2006). There was no need to check for linearity with choice intentions, grade, and gender, because these variables were dichotomy variables that had only linear relationships with other variables (Tabachnick and Fidell, 2006). Furthermore, the correlation between attitude and PBC was significantly positive (*r* = 0.58, *p* < 0.001), which indicated that the two variables had a linear relationship. The results of collinearity diagnostics showed that the VIF among the two measured variables and the demography variables ranged from 1.01 to 1.54, which indicated that no multicollinearity was evident.

**Table 1:** Sample characteristics and descriptive statistics.

Variable	*%*	*M(SD)*	Min	Max	Skewness	Kurtosis
**Dependent variable: choice intention**						
*General learning situation*						
Playing online games	3.96					
Learning	96.04					
*Peer support situation*						
Playing online games	17.17					
Learning	82.83					
*Parental monitoring situation*						
Playing online games	6.60					
Learning	93.40					
**Independent variables**						
Attitude		2.71(1.02)	1.00	5.00	0.24	-0.47
PBC		2.58 (1.04)	1.00	5.00	0.27	-0.66
**Control variables**						
*Grade*						
Grade 5	48.49					
Grade 7	51.51					
*Gender*						
Male	50.57					
Female	49.43					


*PBC, perceived behavioral control.*

Then, the effects of attitude and PBC on adolescents’ choice intention between playing online games and studying were examined. Given that the dependent variables were all dichotomous (participant’s choice intention: 0 = intending to do homework, 1 = intending to play online games), three logistic regressions were conducted in each situation, respectively. Specifically, a logistic regression analysis was performed in the general learning situation, in which the participants’ choice intentions (dependent variable) were regressed on attitude and PBC (independent variables), controlling for grade (0 = Grade 5, 1 = Grade 7) and gender (0 = male, 1 = female). The other two logistic regression analyses for the peer support situation and the parental monitoring situation were the same as the one for the general learning situation. The variables in the models were not standardized or centered. All the three logistic regression models were analyzed with maximum likelihood estimation (ML). Goodness of model fit was evaluated using the Hosmer–Lemeshow test, in which a non-significant chi-square meant that the model was a good fit (Tabachnick and Fidell, 2006). Wald statistics, odds ratios (OR), and 95% confidence intervals of OR were calculated to further understand how attitude and PBC predicted adolescents’ choice intentions. Finally, the differences between the numbers of participants intending to play online games in the three situations were calculated using two Pearson Chi-square tests to examine the effects of manipulated subjective norm on participants’ choice intention.

## Results

### The Effect of Attitude and PBC on Adolescents’ Online Game Choice Intention

The results of descriptive statistics for effect of attitude and PBC on playing online games showed that most participants reported medium levels of positive attitude (*M* = 2.71, *SD* = 1.02) and medium levels of PBC for playing online games (*M* = 2.58, *SD* = 1.04). Furthermore, the results of descriptive statistics for participants’ choice intention showed that there were 21 students (boys: *n* = 14, girls: *n* = 7; Grade 5: *n* = 12, Grade 7: *n* = 9) choosing to play online games in the general learning situation; there were 91 students (boys: *n* = 50, girls: *n* = 41; Grade 5: *n* = 53, Grade 7: *n* = 38) choosing to play online games in the peer support situation; and there were 35 students (boys: *n* = 17, girls: *n* = 18; Grade 5: *n* = 24, Grade 7: *n* = 11) choosing to play online games in the parental monitoring situation. Then, we conducted three logistic regression analyses with demographic variables (grade and gender) as the controlling variables. Attitude and PBC were the independent variables. The participants’ choice intentions in each situation (1 = intending to do homework, 2 = intending to play online games) were the dependent variables.

**FIGURE 1:**
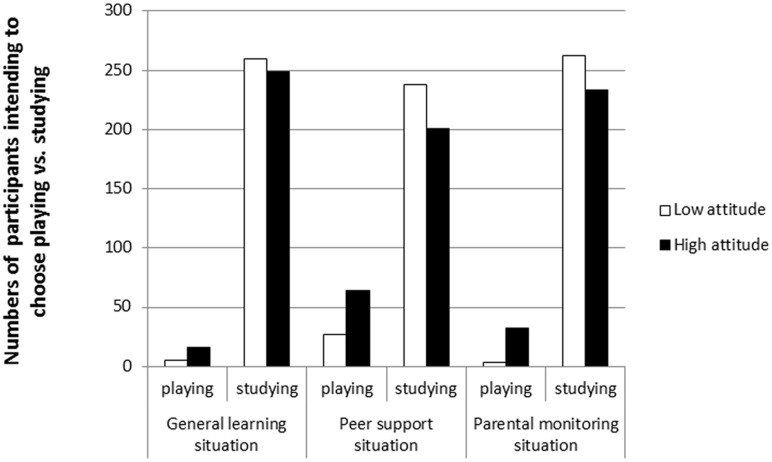
**The choice intention of participants with high attitude and participants with low attitude**.

The results for these models are illustrated in **Table 2**. The results of the Hosmer–Lemeshow test showed that the three logistic regression models were all a good fit (χ^2^ = 7.34, 7.11, and 3.19, respectively, *p* > 0.05). The effects of the controlling variables (gender and grade) were non-significant in the three situations. As predicted, attitude significantly influenced participants’ choice intentions in the three situations (wald: χ^2^ = 5.43, 9.07, and 9.55, respectively, *p* < 0.05; odds ratio: *OR*_general learning situation_ = 2.05, *OR*_peer support situation_ = 1.57, *OR*_parental monitoring situation_ = 2.12). Specifically, positive attitude increased the intention to choose to play online games when participants had a conflicting choice (play online games vs. do homework). The influence of attitude was also observed in the peer support situation and the parental monitoring situation. To illustrate these effects, this study presented the results for participants’ high and low attitude scores based on a median split: There were more participants with high attitude scores (the numbers were 16, 64, and 32, respectively) intending to choose playing online games than ones with low attitude scores (the numbers were 5, 27, and 3, respectively) in the three different situations, whereas there were fewer participants with high attitude scores (the numbers were 249, 201, and 233, respectively) intending to choose doing homework than those with low attitude scores (the numbers were 260, 238, and 262, respectively) in the three different situations (see **Figure 1**).

**Table 2:** Logistic regressions on participants’ choice intention in different situations.

Predictor	*B*	Wald	*p*	*e^B^* (*OR*)	CI 95%
*General learning situation*					
Hosmer–Lemeshow test: χ^2^ = 7.34, *df* = 8, *p* = 0.50					
Standard residual: ranging from -0.98 to 3.05					
Grade	0.04	0.01	0.929	1.04	0.41–2.70
Gender	-0.18	0.12	0.729	0.84	0.31–2.28
Attitude	0.72	5.43	0.020^∗^	2.05	1.12–3.74
PBC	0.73	5.98	0.015^∗^	2.08	1.16–3.74

*Peer support situation*					
Hosmer–Lemeshow test: χ^2^ = 7.11, *df* = 8, *p* = 0.53					
Standard residual: ranging from -1.41 to 2.58					
Grade	-0.32	1.60	0.200	0.73	0.45–1.19
Gender	0.10	0.16	0.697	1.10	0.68–1.82
Attitude	0.45	9.07	0.003^∗∗^	1.57	1.17–2.11
PBC	0.49	11.91	0.001^∗∗^	1.64	1.24–2.17

*Parental monitoring situation*					
Hosmer–Lemeshow test: χ^2^ = 3.19, *df* = 8, *p* = 0.92					
Standard residual: ranging from -1.33 to 2.92					
Grade	-0.61	2.33	0.126	0.54	0.25–1.19
Gender	0.67	2.86	0.092	1.96	0.90–4.28
Attitude	0.75	9.55	0.002^∗∗^	2.12	1.32–3.43
PBC	0.62	7.81	0.005^∗∗^	1.87	1.21–2.90


*^∗∗^*p* < 0.01; ^∗^*p* < 0.05. PBC, perceived behavioral control.*

**FIGURE 2:**
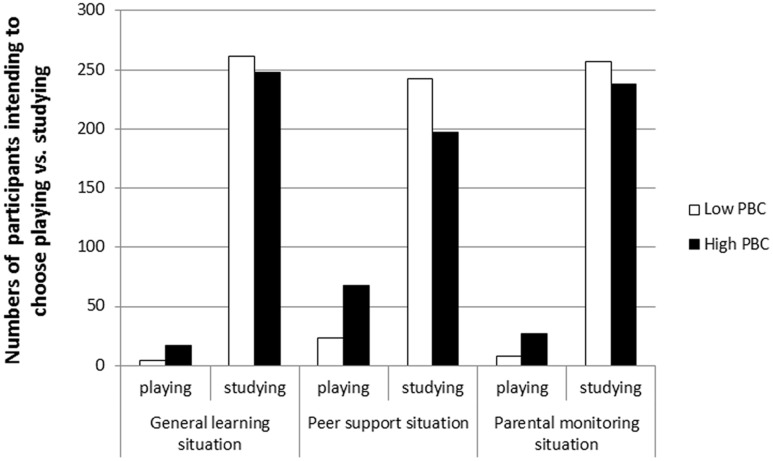
**The choice intention of participants with high PBC and participants with low PBC**.

High PBC scores also significantly contributed to the odds of intending to choose playing online games in the general learning situation, the peer support situation, and the parental monitoring situation (Wald: χ^2^ = 5.98, 11.91, and 7.81, respectively, *p* < 0.05; odds ratio: *OR*_general learning situation_ = 2.08, *OR*_peer support situation_ = 1.64, *OR*_parental monitoring situation_ = 1.87). The results for participants’ high and low scores in PBC based on a median split were as follows: There were more participants high in PBC (the numbers were 17, 68, and 27, respectively) intending to choose playing online games than those low in PBC (the numbers were 4, 23, and 8, respectively) in the three different situations, whereas there were fewer participants with high PBC scores (the numbers were 248, 197, and 238, respectively) intending to choose doing homework than those with low PBC scores (the numbers were 261, 242, and 257, respectively) in the three different situations (see **Figure 2**).

### The Effect of Manipulated Subjective Norm on Adolescents’ Online Game Choice Intention

Two Chi-square tests were conducted to examine the effects of manipulated subjective norm on the participants’ choice intention between the two options (playing online games vs. doing homework). Specifically, the differences between the number of participants intending to choose playing online games in the general learning situation and in the peer support situation were calculated; then, the number of participants intending to choose to play online games in the parental monitoring scenario was also calculated and compared to the peer support scenario. These results showed that manipulated subjective norm had a significant impact on adolescents’ choice intention regarding playing online games.

As shown in **Table 3**, as expected, there were more participants in the peer support situation who intended to choose playing online games than those in the general learning situation [χ^2^(1) = 53.65, *p* < 0.001]. In the parental monitoring situation, the proportion of participants who intended to choose playing online games significantly decreased compared with the peer support situation [χ^2^(1) = 62.10, *p* < 0.001].

**Table 3:** Intention to play online game/do homework in different situations.

Situation	Play online game	Do homework	χ^2^
(1) General learning situation	21 (3.96%)	509 (96.04%)	
(2) Peer support situation	91 (17.17%)	439 (82.83%)	2 ≠ 1^∗∗∗^
(3) Parental monitoring situation	35 (6.60%)	495 (93.40%)	3 ≠ 2^∗∗∗^


*Situation 2 vs. 1, χ^2^ = 53.56, *df* = 1, *Cramer’s V* = 0.32; Situation 3 vs. 2, χ^2^ = 62.10, *df* = 1, *Cramer’s V* = 0.34. ^∗∗∗^*p* < 0.001.*

## Discussion

The present study investigated how and why Chinese adolescents choose between playing online games and completing learning tasks when playing online games would use up learning time, using TPB as theoretical framework, which has been widely used to predict adolescents’ behavior and intention (e.g., Baker and White, 2010; Christiana et al., 2014; Walrave et al., 2014; Tessier et al., 2015). Specifically, the predictive roles of attitude, PBC, and subjective norm in adolescents’ decision making regarding online games, as outlined by TPB, were investigated in this study, wherein a conflict between playing online games and doing homework was introduced. As predicted by H1 and H2, the results showed that the participants with a positive attitude and high PBC toward online gaming were more likely to choose playing online games when facing the conflict (in the general learning situation). These results were consistent with previous research suggesting that attitude and PBC influence participants’ continued intention to play online games (Lee and Tsai, 2010; Hong et al., 2011). In China, primary and secondary school students have to spend a considerable amount of time on learning even after school, and playing online games might consume time originally designated for learning. Thus, academic tasks can represent a “time cost” for them. The results of this study suggested that positive attitude and high PBC toward online games increased the odds of choosing to play online games, even when playing online games led to the time cost.

With regard to the influence of subjective norm, the results of the present study showed that both peer support and parental monitoring, as sub-elements of subjective norm, had an impact on adolescents’ choice intentions toward playing online games and studying. Concerns that traditional measuring of subjective norm might be insufficient when the important others have contradictory views on online gaming, the present study replaced measuring subjective norm by manipulating subjective norm. Specifically, based on the EST’s microsystem, peers and parents were chosen to represent important others in this study. Then, subjective norm was manipulated as perceived peer support and parental monitoring, using different scenarios. In peer support situation, the participants perceived that their peers supported their playing online games. As predicted by H3a, the results showed that peer support significantly increased the odds of intending to choose playing online games versus doing homework. These results were consistent with previous studies, which suggested that peer support positively predicted online game intentions and behaviors (Setterstrom, 2011; Chang et al., 2014). In the parental monitoring situation, participants perceived that their parents objected to their playing online games. Results showed that the number of participants who intended to choose to play online games significantly decreased in this situation, which supported H3b. These results supported the research suggesting that children would respond positively to parental monitoring (Weigle and Reid, 2014). Both of these results provided evidence that subjective norm (perceived peers’ support and parents’ non-support about online gaming) had a significant influence on adolescents’ choice intention about playing online games versus doing homework. In addition, the logistic regression results showed that attitude and PBC could still predict the intention of playing online games in the peer support situation and the parental monitoring situation, which further supported H1 and H2.

### Implications

Adolescents’ online games behavior has been a popular topic of psychological research, including online game addiction, factors influencing online game behavior, and the consequence of playing online games (e.g., Lee and Tsai, 2010; Hellström et al., 2012; Lam et al., 2013; Park et al., 2016; Smohai et al., 2016; Lee and Kim, 2017). This study addressed a relatively underexplored context of online game behavior: how and why Chinese adolescents choose between playing online games and studying, using TPB as theoretical frame. The findings suggested that both attitude and PBC, as outlined by TPB, influenced adolescents’ online gaming choice intention, and the effects were reliable across various contexts (general learning situation, peer support situation, and parental monitoring situation). Moreover, these findings provided evidence that TPB could not only predict the general intention of adolescents to play online games, but also predict their decision-making intention between playing online games and doing homework. In addition, previous studies have cast doubt on the effectiveness of using the subjective norm in predicting individuals’ intention (Terry and Hogg, 1996; Terry et al., 1999; Baker and White, 2010; Chang et al., 2014). One explanation for these disputes may be the insufficient measurement of subjective norm. This study illuminated the effect of subjective norm on online gaming choice intention in a specific common case in China (peers support playing online games whereas parents object to playing online games), by replacing self-reported subjective norm with experimentally manipulating the parent and peer norms.

This study also has important implications for teachers and parents. Certainly, appropriate online gaming has many positive effects on adolescents, such as improving visuospatial cognition, positive affect, and social interactions (Cole and Griffiths, 2007; Ferguson, 2007; Chiang et al., 2011). However, excessive gaming distracts adolescents from academic work (Suhail and Bargees, 2006; Jiang, 2014). Thus, it makes sense to guide adolescents to play online games in a healthy, appropriate manner. The results of this study provide guidance to parents as they help their children avoid excessive online gaming. Specifically, when faced with the choice between playing online games and completing homework, adolescents’ choice intention is determined by their attitude and PBC toward playing the games. In addition, important others, such as parents and peers, influence an adolescent’s choice intention between playing online games and learning.

### Limitations

Although this study provides interesting insights into adolescents’ online gaming behaviors, several limitations should be mentioned. First, this study was conducted against the background of Chinese culture, wherein most adults hold a negative view of online gaming (Liu et al., 2015). However, the positive effect of online gaming has received increasing attention globally (Cole and Griffiths, 2007; Ferguson, 2007; Chiang et al., 2011). Considering the conflicting views about online gaming between different cultures, a cross-cultural study with the same theme (choosing between online gaming and learning) could be an interesting topic for future study. Second, this study created a general learning situation wherein the relation between the predictors emphasized in TPB and decision-making intentions about playing online games was investigated. This effort made the present study more practical, but the situation was not specific enough. In other words, this study only focused on a general situation while disregarding the characteristics of a specific situation (e.g., how important and time-sensitive the homework was). This issue should be addressed in future research. Third, the parental monitoring situation was manipulated after the peer support situation, which might lead to an order effect. Further research needs to repeat the findings of this study by controlling the order effect. For example, future researchers could separate the participants into two groups that encountered the cases in the order of General learning situation → Peer support situation → Parental monitoring situation or in the order of General learning situation → Parental monitoring situation → Peer support situation, respectively. Finally, this study examined the effects of attitude, PBC, and manipulated subjective norm on choice intention between playing online games and doing homework. As posited by TPB (Ajzen, 1991), an individual’s actual actions in a certain case were the direct result of intention. It is important for future work to determine if the three predictors of the TPB can influence the actual choice behaviors between online gaming and learning.

## Author Contributions

JW, R-DL, and YD: analyze data; write manuscript. R-DL, LX, and RZ: collect data.

## Conflict of Interest Statement

The authors declare that the research was conducted in the absence of any commercial or financial relationships that could be construed as a potential conflict of interest.
